# Counteractive effects of antenatal glucocorticoid treatment on D1 receptor modulation of spatial working memory

**DOI:** 10.1007/s00213-016-4405-8

**Published:** 2016-08-23

**Authors:** Kanwar Virdee, Jiska Kentrop, Bianca Jupp, Bethany Venus, Daniel Hensman, Simon McArthur, James Wilkinson, Trevor W. Robbins, Glenda Gillies, Jeffrey W. Dalley

**Affiliations:** 1 Department of Psychology, University of Cambridge, Downing Street, Cambridge, CB2 3EB UK; 2 Behavioural and Clinical Neuroscience Institute, University of Cambridge, Downing Street, Cambridge, CB2 3EB UK; 3 Department of Biomedical Sciences, University of Westminster, New Cavendish Street, London, W1W 6UW UK; 4 Division of Brain Sciences, Imperial College London, Hammersmith Hospital, London, UK; 5 Department of Psychiatry, University of Cambridge, Cambridge, CB2 2QQ UK

**Keywords:** Prefrontal cortex, Dopamine, Dopamine receptors, Memory, Schizophrenia

## Abstract

**Rationale:**

Antenatal exposure to the glucocorticoid dexamethasone dramatically increases the number of mesencephalic dopaminergic neurons in rat offspring. However, the consequences of this expansion in midbrain dopamine (DA) neurons for behavioural processes in adulthood are poorly understood, including working memory that depends on DA transmission in the prefrontal cortex (PFC).

**Objectives:**

We therefore investigated the influence of antenatal glucocorticoid treatment (AGT) on the modulation of spatial working memory by a D_1_ receptor agonist and on D1 receptor binding and DA content in the PFC and striatum.

**Methods:**

Pregnant rats received AGT on gestational days 16–19 by adding dexamethasone to their drinking water. Male offspring reared to adulthood were trained on a delayed alternation spatial working memory task and administered the partial D_1_ agonist SKF38393 (0.3–3 mg/kg) by systemic injection. In separate groups of control and AGT animals, D_1_ receptor binding and DA content were measured post-mortem in the PFC and striatum.

**Results:**

SKF38393 impaired spatial working memory performance in control rats but had no effect in AGT rats. D_1_ binding was significantly reduced in the anterior cingulate cortex, prelimbic cortex, dorsal striatum and ventral pallidum of AGT rats compared with control animals. However, AGT had no significant effect on brain monoamine levels.

**Conclusions:**

These findings demonstrate that D_1_ receptors in corticostriatal circuitry down-regulate in response to AGT. This compensatory effect in D_1_ receptors may result from increased DA-ergic tone in AGT rats and underlie the resilience of these animals to the disruptive effects of D_1_ receptor activation on spatial working memory.

## Introduction

Stress during critical periods of development has widely recognised detrimental effects on maturing neuronal populations in the brain with implications for the aetiology of various clinical disorders (Gillies et al. 2014; Slotkin et al. 2006; Spear 2000). Thus, stress during the perinatal period is thought to play a contributory role in depression, attention-deficit hyperactivity disorder (ADHD), schizophrenia and other psychiatric disorders (Debnath et al. 2015; Khashan et al. 2008; Koenig et al. 2002; Van den Bergh et al. 2005). Findings from structural and functional imaging studies point to multifaceted loci of such disorders, including the limbic corticostriatal systems and abnormalities in dopamine (DA) and serotonin (5-HT) neurotransmission (Castellanos and Tannock 2002; Catts et al. 2013; Spear 2000; Van den Bergh et al. 2005). Indeed, abnormal DA receptor signalling in the prefrontal cortex (PFC) may underlie the impaired working memory performance of schizophrenic patients (Abi-Dargham et al. 2002; Goldman-Rakic et al. 2004).

Convergent research findings in non-human primates and rodents support a role of PFC DA in spatial working memory (Brozoski et al. 1979; Bubser and Schmidt 1990; Simon 1981). PFC-dependent functions depend on the local level of D_1_ receptor activation with low and high levels of D_1_ receptor stimulation impairing cognitive performance (Granon et al. 2000; Mizoguchi et al. 2009; Sawaguchi and Goldman-Rakic 1991; Verma and Moghaddam 1996; Zahrt et al. 1997). Such findings suggest an underlying U-shaped function, consistent with the proposed role of DA acting on D_1_ receptors in modulating the spatial tuning properties of PFC neurons (Sawaguchi et al. 1988; Vijayraghavan et al. 2007; Williams and Goldman-Rakic 1995; Yang and Seamans 1996). Collectively, these findings accord with the view that the PFC is modulated by stress and arousal through D_1_ receptor-dependent mechanisms (Robbins and Arnsten 2009).

We previously reported that antenatal glucocorticoid treatment (AGT) with the synthetic glucocorticoid, dexamethasone, during the late gestational period dramatically increased the population size of midbrain DA neurons in the ventral tegmental area and substantia nigra zona compacta of adult rats (McArthur et al. 2005; McArthur et al. 2007). Dexamethasone was administered non-invasively via the dam’s drinking water on gestational days 16–19. In a recent study, using the same procedure, we found that AGT produced profound, sexually dimorphic changes in markers of DA neurotransmission but had surprisingly negligible effects on several appetitive behaviours known to depend on the mesolimbic DA system, including Pavlovian conditioned approach, psychostimulant-induced locomotor activity and intravenous cocaine self-administration (Virdee et al. 2014). Based on the apparent behavioural resilience in AGT animals, we hypothesised that neural compensatory mechanisms, including a dysregulation of D_1_ receptors in the striatum (Virdee et al. 2014), were sufficient to overcome the expansion of DA neurons in adult rats exposed to dexamethasone in utero.

In the present study, we investigated the hypothesis that cognitive functions that depend on D_1_ receptor mechanisms may be particularly susceptible to AGT. We therefore investigated the performance of AGT rats reared to adulthood on a delayed alternation spatial working memory task, known to be sensitive to disruption by excessive D_1_ receptor activation (Zahrt et al. 1997). We predicted that increased dopaminergic tone in the PFC may lead to compensatory reductions in D_1_ receptors in this region with concomitant effects on the modulation of spatial working memory by a D_1_ receptor agonist.

## Methods

### Subjects

Time-mated female Sprague-Dawley rats (Harlan Olac, Bicester, UK) were kept under a 12/12-h reversed light–dark cycle (lights off at 07.00 h) in holding rooms with controlled temperature (21–23 °C) and humidity (63 %). Standard rat chow and water were available ad libitum*.* Antenatal glucocorticoid treatment was carried out on gestational days 16–19. During this time, sodium dexamethasone phosphate (Faulding Pharmaceuticals, Royal Leamington Spa, UK) was added to the drinking water at a concentration of 0.5 μg/ml. Control dams were given unadulterated drinking water for the entire duration of their pregnancies.

We previously estimated that this procedure for administering dexamethasone delivers a daily dose of approximately 75 μg/kg to the dam (McArthur et al. 2005; McArthur et al. 2006; Theogaraj et al. 2005), a dose comparable to that used in perinatal medicine in cases of threatened premature birth (Jobe and Soll 2004). However, based on previous pharmacokinetic data (Varma 1986), plasma levels of dexamethasone in the developing foetus are likely to be lower than levels that promote foetal lung maturation (Samtani et al. 2006a, b). Since the developing rat brain does not express detectable levels of the multi-drug resistant gene product P-glycoprotein, which extrudes dexamethasone from the brain (Matsuoka et al. 1999), but does express glucocorticoid receptors from embryonic day 15.5 (Diaz et al. 1998), late-gestation dexamethasone is likely to have acted directly in the foetal rat brain.

To offset litter-of-origin effects, the number of male pups taken from each litter to form the experimental groups was limited to two. At 2 months of age, rats were housed in groups of four in each cage. Food was restricted to 15 g of laboratory chow a day (Purina, UK) 1 week prior to the start of behavioural testing. This commenced when animals were 3 months of age. All behavioural testing was performed during the light phase of the light–dark cycle (between 13.00 and 17.00 h). Rats were fed after behavioural testing with a quantity of food sufficient to maintain 85–90 % free feeding weight. All animal procedures were carried out in compliance with the United Kingdom Animals (Scientific Procedures) Act of 1986 and in accordance with local ethical guidelines at Cambridge University.

### Delayed alternation task

A T-maze was used to assess delayed alternation in control and AGT rats. The maze was elevated 50 cm above the floor and constructed from wood with a stem arm and two side goal arms, bounded by a perimeter ledge 2 cm high to prevent animals falling from the apparatus. A food well was located at the end of each goal arm. The goal arms were separated by a fixed central partition that extended 25 cm into the start arm. During information trials, a wooden block 2-cm high was used to block one of the goal arms. Hot water was used to clean the apparatus prior to testing each animal.

Following habituation during a single 20-min session where rats could freely explore the apparatus, subjects underwent alternation shaping, in which one goal arm was blocked, thus forcing the rat to enter and consume a reward pellet from the open arm (an information run). Once the rat had consumed the reward pellet, it was gently picked up and immediately returned to the starting point again for a second run (choice run) in which free access was allowed to both goal arms but with only the previously blocked arm being baited with reward. Alternation shaping without delay consisted of 10 trials with sessions conducted daily for 14 consecutive days. Each trial was independent and consisted of two phases: a rewarded forced run followed by a free choice alternation run. Each session consisted of an equal number of pseudorandom forced entries into the left and right goal arms, with no more than two consecutive forced entries into the same arm. A correct choice was scored if the rat entered the baited (previously blocked) arm in the choice run and consumed the reward pellet, whereas an error was scored if the rat visited the same arm in the choice run as that entered in the forced trial. Any rat failing to make the information run within 45 s was removed from the T-maze and tested again. Training was continued in this manner until the rats attained a stable level of performance. The latency from start arm to the consumption of the reward pellet for both the information run and choice run (whether correct or incorrect) was recorded using a digital stopwatch by an observer blind to the experimental groups.

On completion of the forced alternation training, rats were trained on a discrete, paired trial version of the delayed alternation task. Delayed alternation testing was identical to alternation shaping except that a delay period of 90 s was interposed between the information run and the choice run. During the delay interval, the rat was returned to a holding cage containing three other cage mates that were being tested on the same day. The delay or retention interval was decided on the basis of preliminary validation experiments that revealed that delays of 30 or 60 s were insufficient to produce reliable deficits in alternation performance. In pilot studies, we also established that a dose of 0.3 mg/kg SKF38393 was insufficient to modulate performance on this task in control and AGT animals.

### Drug administration

The partial D_1/5_ receptor agonist SKF38393 was purchased from TOCRIS Biosciences (UK) and dissolved in 0.9 % saline. It was administered by intraperitoneal injection (1 ml/kg) at doses of 1 and 3 mg/kg using a randomised Latin square design. Each rat underwent pre-injection runs for which there were 12 trials given in a random order (3 left blocks and 3 right blocks with no delay; 3 left blocks and 3 right blocks with a 90-s delay). Thereafter, rats were injected with vehicle or SKF38393 and returned to their holding cages for 30 min. They were then tested on the T-maze during 12 post-injection trials, as described above. Each dose of SKF38393 (or vehicle) was administered after a 48-h washout period. Since SKF38393 produced no discernible effect on delayed alternation in AGT rats, unlike controls, we also tested the effects of a higher dose of this compound (10 mg/kg) in AGT animals.

### Open-field locomotor activity

The open-field test was used to measure spontaneous locomotor activity. The test chamber (San Diego Instruments, USA) was fitted with infrared photocell beams with the following dimensions: 40 cm (*W*) × 40 cm (*D*) × 37.5 cm (*H*). Animals were first habituated to the apparatus on two consecutive daily sessions, with each session lasting 90 min. The effects of SKF38393 (and saline) were assessed the next day with beam breaks recorded in bins of 5 min over a 90-min period, using a randomised Latin square design and a 48-h washout period. SKF38393 was administered by intraperitoneal injection at 1, 3, and 10 mg/kg in a volume of 1 ml/kg, 30 min before the animals were placed in the open-field apparatus.

### Receptor autoradiography

Control and AGT rats were sacrificed by decapitation and their brains removed and prepared for quantification of D1 receptors using the D_1/5_ ligand [^3^H]-SCH23390, according to a previously published protocol (McArthur et al. 2007). Autoradiographic films were developed and converted to digital form using an MCID Core system attached to a CoolSNAPPro_cf_ camera (Interfocus Imaging Ltd., Cambridge, UK). Systematic random sampling within the specified brain regions was performed for densitometric analysis, and average values from six distinct brain sections per region per rat were used to obtain group means. Regions of interest included the anterior cingulate cortex (ACg), prelimbic cortex (PrL), infralimbic cortex (IL), dorsal striatum (dST), ventral striatum (vST: core and shell combined) and the ventral pallidum (vPal).

### Post-mortem analysis of monoamines

Snap-frozen brains were sliced on a JungCM3000 cryostat (Leica Microsystems Ltd., Milton Keynes, UK) into 150-μm-thick sections. These were thaw-mounted onto glass slides and circular micro-punches of 0.75 mm in diameter were taken bilaterally from the infralimbic cortex, prelimbic cortex, cingulate cortex, orbitofrontal cortex, nucleus accumbens core, nucleus accumbens shell and dorsal striatum. Tissue aliquots were stored in vials at −80 °C until further processing. At the time of processing, they were thawed and homogenised in 75 μl of 0.2 M perchloric acid using a hand-held mechanised pellet pestle (Kimble-Kontes, Vineland, NJ, USA). The tissue suspensions were then centrifuged at 6000 rpm for 10 min at 4 °C. DA, NA and 5-HT were determined by reversed phase high-performance liquid chromatography (HPLC) with electrochemical detection. In each case, 25 μl of sample was injected onto a C18 ODS 3-μm analytical column (100 mm length × 4.6 mm i.d., Hypersil Elite, Phenomenex, UK) with a mobile phase (citric acid 31.9 g/L, sodium acetate 2.0 g/L, 1-octanesulfonic acid 460 mg/L, EDTA 30 mg/L and methanol 150 ml/L) delivered at 0.8 ml/min. Monoamines were quantified using an ESA Coulochem II detector and an analytical cell (ESA model 5014) with two electrodes in series. The potential of the first (reducing) electrode was held at −200 mV, while the potential of the second (oxidising) electrode was set to +250 mV relative to a platinum reference electrode. The resultant signal from the second electrode was integrated using Dionex Chromeleon software. Neurochemical levels were calculated relative to external standards and expressed as picomole per milligram of wet tissue weight.

### Statistical analysis

Behavioural data were analysed using repeated-measures ANOVA (SPSS, version 21, IBM) with delay (two levels: no delay; 90 s) and SKF38393 dose (three levels: vehicle, 1 and 3 mg/kg) as within-subject’s factors and group (two levels: control and AGT) as a between-subject’s factor. Acquisition data (% correct) were analysed with day (14 levels) and group (2 levels) as the within- and between-subject’s factors, respectively. Significant interactions between factors were analysed further by ANOVA and post hoc Dunnett’s tests, where appropriate. Mauchly’s test of sphericity was applied and the degrees of freedom adjusted using the Huynh-Feldt epsilon when the assumption of sphericity was violated. Autoradiography and HPLC data were analysed by Student’s *t* tests comparing averaged values from various brain regions of AGT male rats to the values obtained from corresponding brain regions of control rats. Data were pooled across the left and right hemispheres. A criterion level of *α* = 0.05 was used to interpret main effects, interactions and post hoc tests.

## Results

### Spatial delayed alternation

**Fig. 1:**
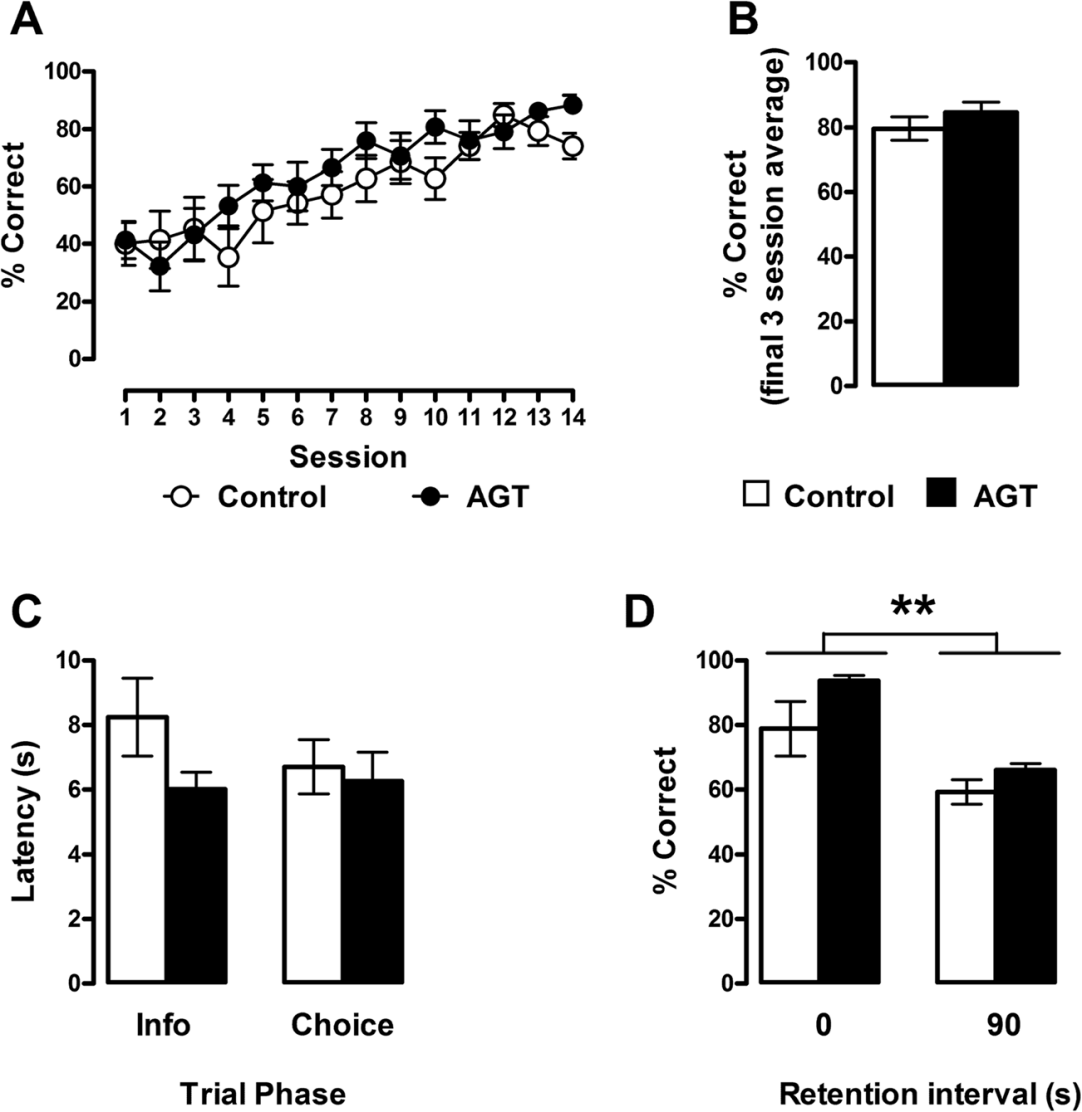
Discrete paired trials forced alternation T-maze task. **a** Acquisition of the forced alternation task in AGT males (*closed symbols*) and control males (*open symbols*) expressed as a percent correct score. Data are means ± 1 SEM (*n* = 14 control males and *n* = 15 AGT males). Repeated-measures ANOVA revealed a significant main effect of session (*F*_(13, 351)_ = 14.74, *p* < 0.01) with no significant between-group difference in percent correct alternation score. **b** Percent correct accuracy averaged over the last three training sessions (sessions 12–14) of forced alternation training. Data are means ± 1 SEM of correct scores expressed as a percentage of 30 paired trials. **c** Latencies to complete the forced information run and the correct choice run during the final three training sessions. AGT males (*closed bars*); control males (*open bars*). **d** Percent choice accuracy following the interposition of a 90-s delay interval between the information and choice runs. Both groups exhibited a diminished accuracy of performance following the long retention interval (**p < 0.01). Control group (*n* = 6); AGT group (*n* = 7)

The acquisition and performance of control (*n* = 12) and AGT (*n* = 14) rats on the delayed alternation spatial working task are shown in Fig. 1. Choice accuracy during forced alternation training increased progressively during each daily session (session: *F*_(13, 351)_ = 14.735, *p* < 0.01: Fig. 1a); however, this improvement was no different between control and AGT rats (group: *F*_(1, 27)_ = 0.858, *p* = 0.362). Indeed, both groups of animals attained comparable and stable levels of accuracy of approximately 80 % by the last 3 days of training (control 79.5 ± 3.6 % correct; AGT 84.6 ± 3.1 % correct, *p* = 0.289: Fig. 1b). There were also no statistically significant differences in response latencies between control and AGT rats during the information and choice trials (Fig. 1c) with averaged latencies for the information and choice trials of 8.2 ± 1.2 and 6.7 ± 0.8 s and 6.0 ± 0.5 and 6.3 ± 0.9 s for control and AGT rats, respectively. During delayed alternation trials where a 90-s delay was imposed between the forced and choice run trials, performance accuracy declined significantly compared with the zero delay condition (*F*_(1, 11)_ = 62.38, *p* < 0.001: Fig. 1d). However, there was no significant main effect of group or group × delay interaction indicating that at baseline AGT had no significant effect on spatial working memory in the delayed alternation task.

### Effect of SKF38393 on delayed alternation performance

**Fig. 2:**
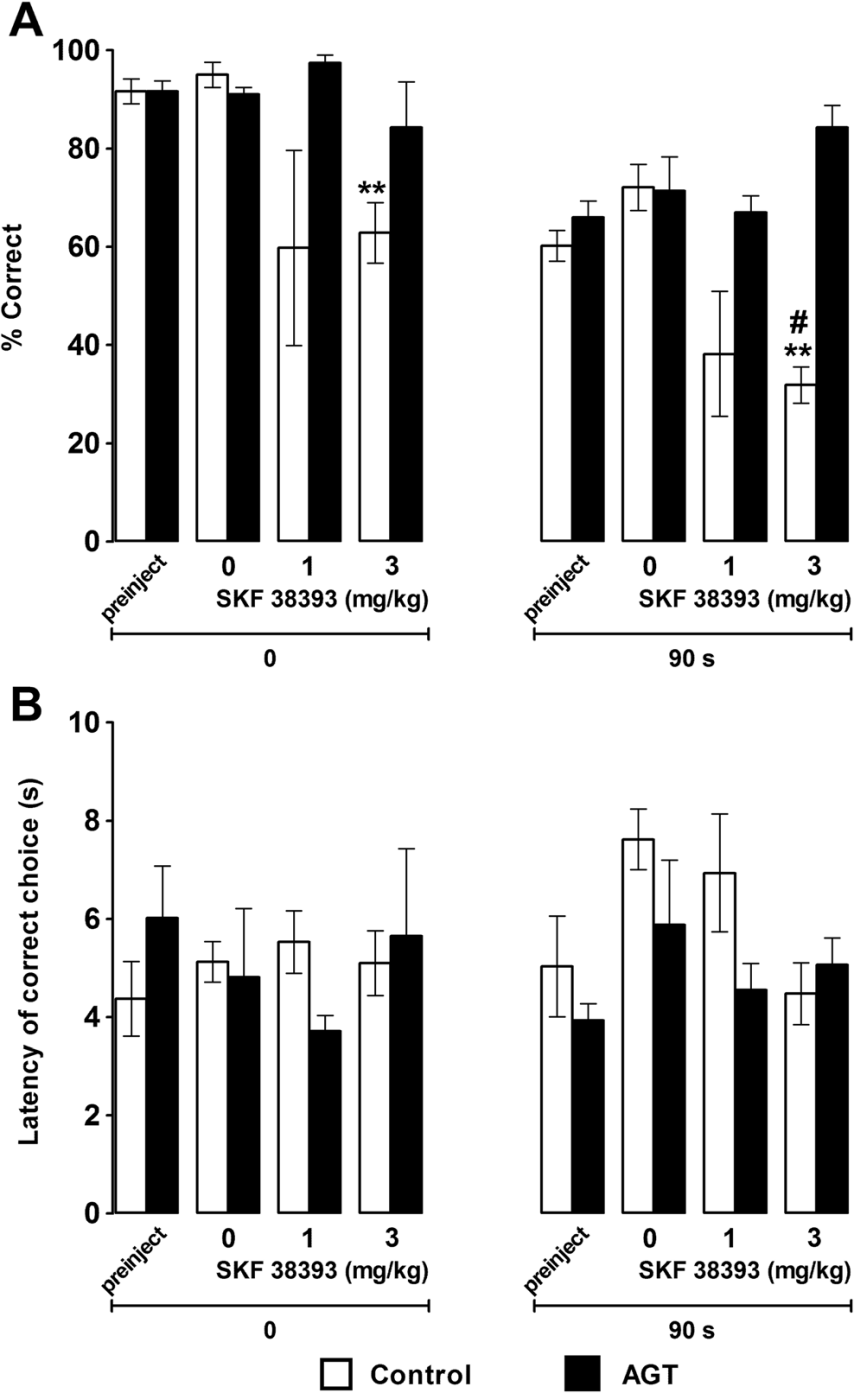
Effect of D1/5 receptor activation on delayed alternation performance. **a** Control (*n* = 5; *open bars*) and AGT (*n* = 5; *closed bars*) rats were pre-treated with 0.9 % saline or the D1/5 receptor agonist SKF38393 30 min before being tested on the T-maze alternation task with trials consisting 0- or 90-s delays. Data are group means ± 1 SEM. **p < 0.01 (vs 0 mg/kg/saline); #*p* < 0.05 (control vs AGT). **b** Lack of effect of SKF38393 on response latencies for correct choice trials before and after the 90-s retention interval (90 s). A baseline level of performance was first established by testing rats on the T-maze before systemic injections of saline or SKF38393 (‘preinject’). Data are group means for control (*n* = 5; *open bars*) and AGT (*n* = 5; *closed bars*) rats

Figure 2 summarises the effects of the D_1/5_ receptor agonist SKF38393 on the delayed alternation task. A global analysis of choice accuracy following SKF38393 administration revealed a main effect of retention interval (*F*_(1, 8)_ = 86.83, *p* < 0.01), a significant interaction between group and SKF38393 dose (*F*_(3, 24)_ = 4.26, *p* = 0.044) and a significant interaction between group, SKF38393 dose and retention interval (*F*_(3, 24)_ = 3.81, *p* = 0.023). Pair-wise comparisons during the 90-s delay period showed that relative to the vehicle control group the highest dose of SKF38393 (3 mg/kg) significantly impaired choice accuracy in control animals but not AGT animals (*p* < 0.01 vs saline; *p* < 0.01 control vs AGT). By contrast, this dose of SKF38393 produced a trend significant improvement in the choice accuracy of AGT rats (*p* = 0.081 vs saline; *p* = 0.025 vs pre-injection baseline). Distinct from AGT rats, SKF38393 also impaired choice accuracy in control rats during the zero delay condition reaching significance at the 3-mg/kg dose level (*p* < 0.01 vs saline). As SKF38393 had no detectable effect on the behavioural performance of AGT rats, we also injected a higher dose of this compound (10 mg/kg). SKF38393 was again without effect in AGT rats (choice accuracy ± SEM, 90 s delay = 84 ± 4 %) but impaired performance in control rats with many subjects failing to complete a single correct trial. These observations indicate that spatial working memory in AGT rats is remarkably resistant to disruption by a D_1/5_ receptor agonist compared with control rats. In addition, global analysis of correct choice latencies following SKF38393 administration revealed no significant main effects or interactions of drug treatment, retention interval or group (Fig. 2b).

**Fig. 3:**
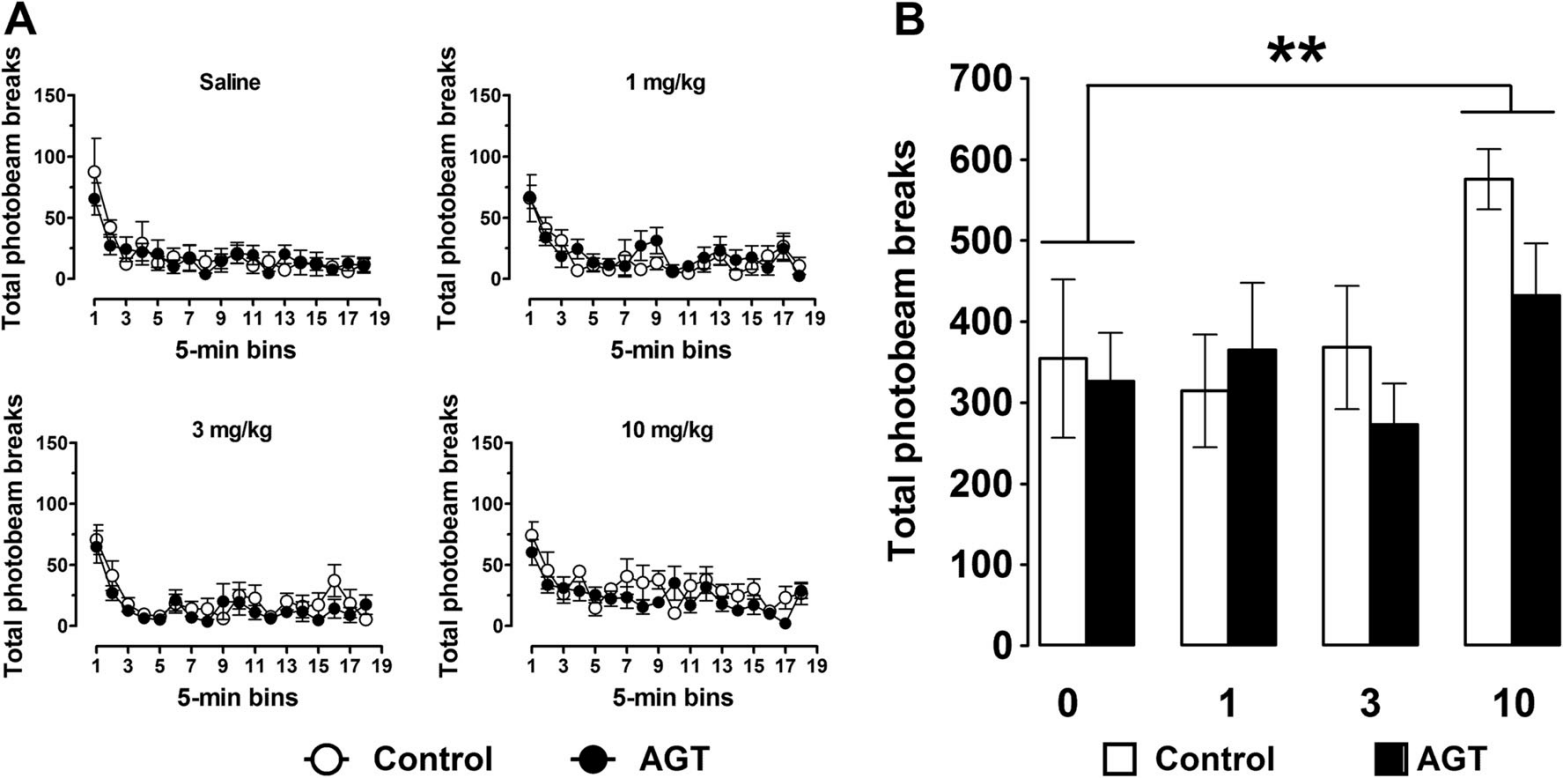
Effect of systemic SKF38393 on open-field locomotor activity. **a** Ambulatory locomotor activity in control *n* = 5; *open bars*) and AGT (*n* = 5; *closed bars*) rats following saline and increasing doses of the D1/5 receptor agonist SKF38393. Data are means ± 1 SEM. **b** Locomotor activity averaged over the 90-min testing period. ANOVA indicated a main effect of dose (*F*_(3, 30)_ = 4.405), *p* = 0.012) and a significant difference (**p < 0.01) between rats injected with 10 mg/kg SKF38393 and saline (control and AGT rats combined)

We next investigated whether the impairing effects of SKF38393 on spatial working memory performance in control rats were due to nonspecific behavioural processes. To examine this possibility, we assessed the effects of SKF38393 on open-field locomotor activity in control and AGT rats. Figure 3a shows the temporal profile of locomotor activity in animals injected with SKF38393. The main analysis revealed a main effect of dose (*F*_(3, 30)_ = 4.41, *p* = 0.012) and time (*F*_(17, 170)_ = 17.35, *p* < 0.001) but no main effect of group or significant interactions. Collapsing the data as a function of dose (Fig. 3b) revealed that a dose of 10 mg/kg SKF38393 produced a mild stimulant effect compared with vehicle-treated animals (*p* = 0.008). Notably, however, lower doses of SKF38393, which impaired working memory performance in control animals, did not differentially affect locomotor activity relative to the AGT group.

### D_1_ receptor autoradiography and post-mortem neurochemistry

**Fig. 4:**
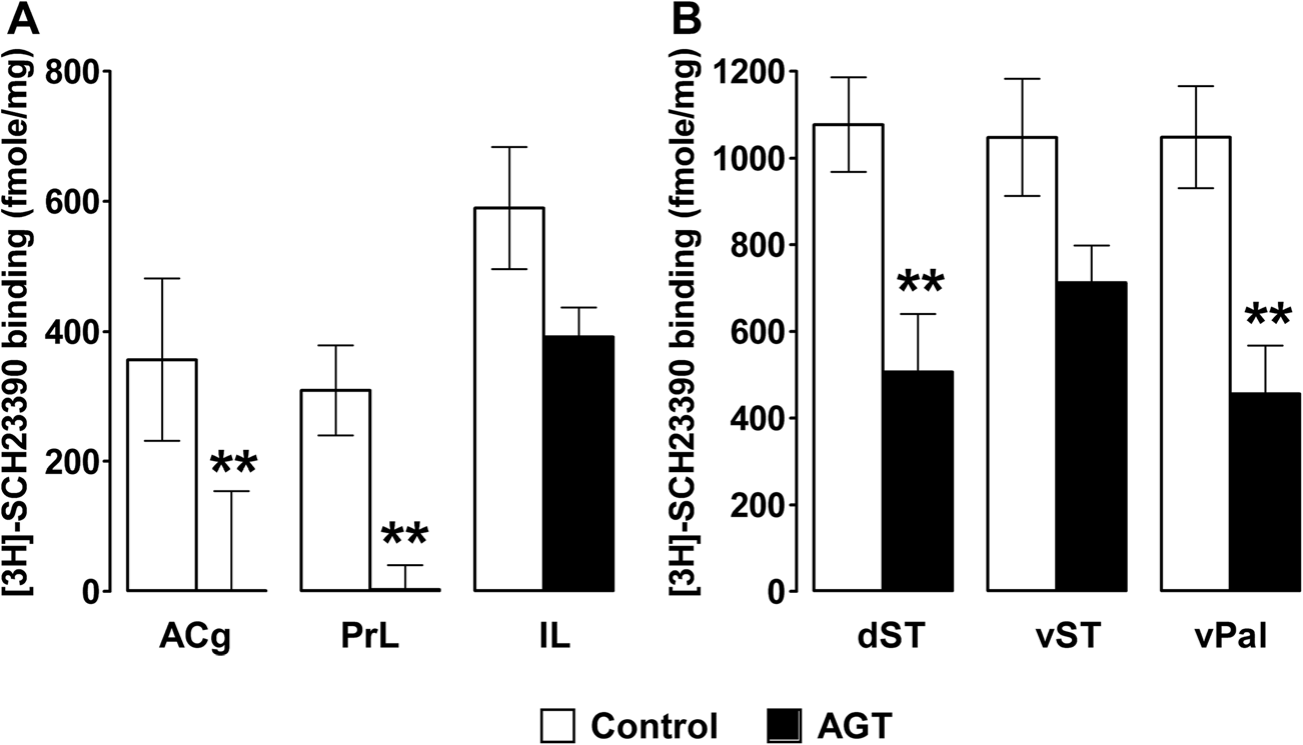
D1 receptor autoradiography. Quantitative comparison of radioligand binding to D1-type receptors in the anterior cingulate cortex (*ACg*), prelimbic cortex (*PrL*), infralimbic cortex (*IL*), dorsal striatum (*dST*), ventral striatum (*vST*) and ventral pallidum (*vPal*) in control (*n* = 6; *open bars*) and AGT (*n* = 6; *closed bars*) rats. Data are means ± 1 SEM. **p < 0.01 (control vs AGT)

Binding densities of [^3^H]-SCH23390 in the medial PFC, striatum and ventral pallidum are shown in Fig. 4a, b. AGT produced a significant reduction in D_1/5_ receptor binding compared with control adult rats in the majority of regions assayed (all *p* < 0.01) except the infralimbic cortex (*p* = 0.088) and ventral striatum (*p* = 0.063). However, levels of the monoamines (NA, DA and 5-HT) and primary metabolites (DOPAC and 5-HIAA) were not significantly affected by antenatal dexamethasone exposure in any of the brain regions examined. In addition, DA or 5-HT turnover rates were not significantly affected by this manipulation (Table 1).

**Table 1:** Post-mortem monoamine and metabolite levels

	DA		NA		5-HT		DOPAC		5-HIAA		DA turnover		5-HT Turnover	
	Control	AGT	Control	AGT	Control	AGT	Control	AGT	Control	AGT	Control	AGT	Control	AGT
IL	1.32 ± 0.63	1.40 ± 0.64	1.45 ± 0.50	1.05 ± 0.22	0.10 ± 0.01	0.18 ± 0.07	5.48 ± 2.13	1.43 ± 0.67	0.71 ± 0.25	0.53 ± 0.22	6.54 ± 3.76	2.68 ± 2.14	6.72 ± 2.44	3.96 ± 2.15
PrL	1.61 ± 0.36	1.24 ± 0.44	1.08 ± 0.11	0.98 ± 0.09	0.14 ± 0.06	0.08 ± 0.02	1.97 ± 0.58	0.42 ± 0.08	0.26 ± 0.07	0.13 ± 0.02	1.22 ± .34	1.40 ± 1.17	2.81 ± 1.08	2.02 ± 0.38
ACg	0.87 ± 0.49	0.90 ± 0.29	1.55 ± 0.64	0.66 ± 0.11	0.27 ± 0.21	0.10 ± 0.04	1.41 ± 0.76	0.35 ± 0.23	0.13 ± 0.08	0.06 ± 0.02	2.04 ± 1.50	0.85 ± 0.73	1.53 ± 0.93	1.03 ± 0.56
OFC	0.51 ± 0.13	0.71 ± 0.18	1.24 ± 0.48	0.67 ± 0.05	0.07 ± 0.01	0.07 ± 0.01	1.38 ± 0.94	0.45 ± 0.30	0.48 ± 0.26	0.26 ± 0.15	2.04 ± 1.26	1.25 ± 1.08	6.88 ± 3.61	4.29 ± 2.52
NAcbC	2.15 ± 0.90	0.79 ± 0.26	2.40 ± 1.42	1.07 ± 0.51	0.11 ± 0.02	0.13 ± 0.02	13.12 ± 7.03	10.84 ± 2.85	1.96 ± 0.29	0.96 ± 0.17	10.62 ± 4.21	15.83 ± 3.18	19.26 ± 3.51	8.08 ± 1.39
NAcbS	2.04 ± 0.52	1.15 ± 0.25	1.17 ± 0.48	1.94 ± 1.20	0.30 ± 0.07	0.15 ± 0.04	13.17 ± 3.78	9.31 ± 4.35	2.05 ± 0.64	1.24 ± .51	7.35 ± 2.04	8.27 ± 2.97	7.18 ± 2.33	7.64 ± 1.50
dST	1.10 ± 0.40	0.46 ± 0.11	0.73 ± 0.23	0.61 ± 0.09	0.12 ± 0.03	0.13 ± 0.03	7.69 ± 3.08	5.53 ± 1.94	4.34 ± 2.68	3.78 ± 1.52	8.71 ± 4.04	13.35 ± 4.07	31.32 ± 12.09	32.81 ± 10.35
dHipp	0.39 ± 0.18	0.75 ± 0.31	1.93 ± 0.84	1.29 ± 0.28	0.07 ± 0.01	0.12 ± 0.04	1.66 ± 0.89	0.31 ± 0.14	0.53 ± 0.29	0.19 ± 0.10	8.53 ± 5.42	0.50 ± 0.23	7.77 ± 3.81	1.40 ± 0.40
vHipp	0.27 ± 0.07	0.63 ± 0.14	1.69 ± 0.36	1.31 ± 0.24	0.04 ± 0.01	0.06 ± 0.01	4.35 ± 2.46	0.43 ± 0.06	0.44 ± 0.26	0.29 ± 0.08	12.67 ± 5.46	0.86 ± 0.26	12.04 ± 5.40	4.88 ± 1.20

Regional brain levels of NA, DA, 5-HT, DOPAC and 5-HIAA in control and AGT rats (each group *n* = 4). Data are means ± SEM (pmol/mg wet weight of tissue). DA and 5-HT turnover is also shown (DOPAC/DA and 5-HIAA/5-HT, respectively)

*IL* infralimbic cortex, *PrL* prelimbic cortex, *ACg* anterior cingulate cortex, *OFC* orbitofrontal cortex, *NAcbC* nucleus accumbens core, *NAcbS* nucleus accumbens shell, *dST* dorsal striatum, *dHipp* dorsal hippocampus, *vHipp* ventral hippocampus

## Discussion

We report the novel finding that rats exposed to AGT are resilient as adults to the disruptive effects of a D_1/5_ receptor agonist on the performance of a spatial working memory task. Dopamine D_1/5_ receptor activation appeared, if anything, to facilitate the retention of working memory in this group of animals, which corresponded with diminished D_1/5_ receptor binding in the anterior cingulate cortex, prelimbic cortex, dorsal striatum and ventral pallidum. Although D_1/5_ receptor dysregulation presumably resulted in part from sustained perturbations in mesencephalic DA neurotransmission, we observed no significant alterations in monoamine levels and turnover in the PFC and striatum. Our findings demonstrate that AGT induces long-lasting abnormalities in the modulation of spatial working memory by D_1_ receptors. Theoretically, our results are consistent with a rightward shift in an underlying U-shaped function regulating D_1_ receptor spatial processing, possibly mediated within the PFC as a consequence of AGT (Robbins and Arnsten 2009; Vijayraghavan et al. 2007). However, since SKF38393 also impaired choice accuracy in control rats during the zero delay condition, this compound may have disrupted aspects of working memory performance separate from mnemonic processing per se.

We reported previously that late gestational exposure to dexamethasone increases the density of DA inputs to the ventral and dorsal striatum and causes profound changes in the regulation of DA release and D_1_ and D_2_ receptors in these regions (Virdee et al. 2014). These neurobiological changes were often sexually dimorphic and were not accompanied by differential effects on a broad range of psychomotor and appetitive behaviours known to depend on the mesolimbic DA system. We explained these findings by enduring molecular adaptations within the subcortical DA systems that we hypothesised were sufficient to compensate for the pronounced increase in DA inputs in the striatum of AGT rats. In the present study, we probed the mesocortical DA system using a delayed alternation spatial working memory task (Bubser and Schmidt 1990). We based our experimental approach on an earlier report showing excessive D_1_ receptor stimulation in the PFC to disrupt spatial working memory performance in rats (Zahrt et al. 1997). Specifically, infusions of the full D_1_ receptor agonist SKF81297 into the dorsal PFC produced a dose-related impairment in spatial delayed alternation; this effect was blocked by the D_1_ receptor antagonist SCH23390, which itself only impaired performance at higher doses. Interestingly, SKF81297 was more disruptive to spatial working memory when infused in the prelimbic cortex than more anterior sites. As acknowledged by the authors, infusions of SKF81297 at this anterior–posterior level probably diffused to adjacent sites as well, including the infralimbic cortex. Indeed, our own analysis found that localised infusions of a D_1_ antagonist in the prelimbic cortex track dorsally to involve the anterior cingulate cortex (Granon et al. 2000). Thus, the precise brain locus in rats underlying the modulation of spatial delayed alternation by D_1_ receptor compounds is unclear. However, the magnitude of dopamine release in the medial PFC, involving mainly the prelimbic cortex, was found to predict the accuracy of memory retrieval on a spatial delayed response task (Phillips et al. 2004).

In the present study, AGT rats were resistant to the disruptive effects of SKF38393 on spatial delayed alternation. AGT animals continued to show high levels of choice accuracy even at the high dose of 10 mg/kg, which strongly impaired performance in control animals. Parsimoniously, this resilience to excessive D_1_ receptor stimulation may be a consequence of down-regulated D1 receptors throughout the forebrain of AGT rats. As D_1_ receptors are expressed on neurons intrinsic to fronto-striato-pallidal circuitry, postsynaptic to dopamine inputs (Strange et al. 1983), the down-regulation in D_1_ receptors we observed may have been driven by a compensatory response to the expansion of midbrain dopamine neurons in AGT rats (McArthur et al. 2005). However, the precise mechanism coupling the presumed increase in dopamine activity in the PFC and D_1_ receptor dysregulation is unclear since in an earlier study we found that dopamine release in the ventral striatum was no different between control and AGT rats, despite D_1_ receptors also being down-regulated in this region (Virdee et al. 2014). This may reflect the tight homeostatic control over dopamine release in this region and perhaps also the PFC, but a further study would be needed to directly monitor dopamine release in the PFC, both under basal and task-related conditions. This is warranted as the ex vivo measures used in the present study were presumably insufficiently sensitive to detect differences in dopamine function in the various regions investigated. However, a previous study reported a small (approximately 10 %) increase in dopamine concentration in the adult rat cerebral cortex following AGT by daily subcutaneous injections of dexamethasone at gestational days 17 to 19 (Slotkin et al. 2006). Nevertheless, it does appear that neurochemical and behavioural deficits only overtly manifest in AGT rats following acute provocation of the dopamine systems. Thus, systemic injections of d-amphetamine increased striatal dopamine release to a significantly greater extent in AGT rats than control rats (Virdee et al. 2014). Similarly, in the present study, behavioural differences between control and AGT rats only emerged when D_1_ receptors were directly activated. These findings suggest that adaptive variations within the dopamine systems, induced by AGT, are especially vulnerable to acute perturbations by selective pharmacological agents.

There are several limitations to our work that merit discussion. Firstly, the receptor binding studies were conducted in a separate cohort of rats to those used in the behavioural pharmacology experiments. Further studies are needed to investigate whether behavioural training and differing periods of food restriction altered the behavioural effects of SKF38393, as suggested by other studies (Carr et al. 2003; Haberny et al. 2004). Secondly, only males were tested in the present study, primarily to circumvent the complication of variations in hormones during the oestrous cycle. It is therefore unclear whether the present findings would generalise to females. This is important as there is strong evidence that prenatal stress and overexposure to glucocorticoids leads to diverse sexually dimorphic effects on the developing brain (Hiroi et al. 2016; McArthur et al. 2007; McArthur et al. 2016; Zuloaga et al. 2011; Zuloaga et al. 2012). Thirdly, it is possible that SKF38393 exerted off-target effects at non-dopaminergic receptors. However, unlike other D_1_ receptor-selective phenyl-benzazepines (e.g. SCH23390), SKF38393 has a low affinity for 5-HT receptors (Neumeyer et al. 2003), suggesting that the effects of SKF38393 in the present study were most likely mediated by D_1/5_ receptors. Nevertheless, further studies would be needed to investigate local effects of D1 receptor activation in the PFC, including possible cognitive enhancing effects on spatial working memory (Chudasama and Robbins 2004). Finally, it is possible that AGT overtly altered maternal pup-directed behaviour. However, this appears unlikely since a concentration of dexamethasone almost twice as high as that used in the present study had no detrimental effects on maternal pup behaviour (Hauser et al. 2006).

Although our results replicate earlier findings showing excessive D_1_ receptor stimulation to impair spatial working memory performance (Zahrt et al. 1997), we were unable to definitively show that SKF38393 disrupts performance in a delay-dependent manner since retention accuracy also declined during the zero delay condition. However, this may reflect the fact that a zero delay is nominal and difficult in practice to achieve using our non-automated delayed alternation task. The real delay was likely to be in the order of several seconds, and this would accord with the shortest delay of 5 s used by Zahrt et al. but still on a timescale known to engage PFC mechanisms (Lapish et al. 2009). Further, SKF38393 produced no obvious differential effects on either open-field locomotor activity or response latencies on the delayed alternation task suggesting that this compound was not generally disruptive to behavioural output. Nevertheless, further studies would be necessary to investigate the mechanism underlying the impairing effects of SKF38393 on delayed alternation performance in control rats, which may involve effects on attention (Passetti et al. 2003), the encoding and temporary storage of information, to the retrieval and flexibility of trial-unique information (Mizumori et al. 1987).

As well as affecting central dopamine pathways, prenatal glucocorticoid exposure exerts a wide spectrum of effects on the developing brain (Matthews 2001). Studies in rodents and primates have shown that foetal glucocorticoid exposure and prenatal stress both cause marked abnormalities in gene expression, receptor composition and structural markers in the hippocampus and PFC (Berger et al. 2002; Uno et al. 1994; Uno et al. 1990). Thus, the resistance of AGT rats to the impairing effects of SKF38393 on working memory performance may be mediated by mechanisms separable from effects on dopamine neurotransmission in the PFC. For example, AGT has been shown to alter serotonin turnover, receptor binding and transporter function in a dose- and region-specific manner (Slotkin et al. 2006; Slotkin and Seidler 2010). Additionally, changes in astro-glial morphology have been implicated in hippocampal remodelling following AGT (McArthur et al. 2016; Shende et al. 2015). Since intra-hippocampal infusions of D1/D5 receptor agonists improve performance on radial maze working memory tasks (Packard and White 1991) that depend on the hippocampus (Spowart-Manning and van der Staay 2004), it is possible that our findings were influenced by drug interactions in the hippocampus. It is also possible that different processes were recruited for optimal task performance (e.g. attention and other executive functions) and that these processes were differentially susceptible to modulation by SKF38393.

In conclusion, the present study shows that excess glucocorticoid exposure during the late gestational stage decreases the sensitivity of rats to a D_1_ receptor agonist on a delayed alternation working memory task. Our results are consistent with a rightward shift in a hypothetical “U”-shaped function underlying the assumed modulation of this task by PFC dopamine. Our findings highlight the profound consequences for brain development of prenatal stress hormones and suggest a mechanism whereby AGT animals may be resilient to stress and anxiogenic stimuli as adults, which generally impair delayed alternation performance (Arnsten 1997; Murphy et al. 1996a; Murphy et al. 1996b; Sahakian et al. 1985). Finally, and more speculatively, our findings may be relevant to the variability of therapeutic drug responses in various neurodevelopmental brain disorders (Hermens et al. 2005).
